# A Blockchain-Based Distributed Paradigm to Secure Localization Services †

**DOI:** 10.3390/s21206814

**Published:** 2021-10-13

**Authors:** Roberto Saia, Alessandro Sebastian Podda, Livio Pompianu, Diego Reforgiato Recupero, Gianni Fenu

**Affiliations:** Department of Mathematics and Computer Science, University of Cagliari, Palazzo delle Scienze, Via Ospedale 72, 09124 Cagliari, Italy; sebastianpodda@unica.it (A.S.P.); pompianu.livio@unica.it (L.P.); diego.reforgiato@unica.it (D.R.R.); fenu@unica.it (G.F.)

**Keywords:** Internet of Things, Internet of Entities, mobile network, blockchain, distributed ledger, localization

## Abstract

In recent decades, modern societies are experiencing an increasing adoption of interconnected smart devices. This revolution involves not only canonical devices such as smartphones and tablets, but also simple objects like light bulbs. Named the Internet of Things (IoT), this ever-growing scenario offers enormous opportunities in many areas of modern society, especially if joined by other emerging technologies such as, for example, the blockchain. Indeed, the latter allows users to certify transactions publicly, without relying on central authorities or intermediaries. This work aims to exploit the scenario above by proposing a novel blockchain-based distributed paradigm to secure localization services, here named the Internet of Entities (IoE). It represents a mechanism for the reliable localization of people and things, and it exploits the increasing number of existing wireless devices and blockchain-based distributed ledger technologies. Moreover, unlike most of the canonical localization approaches, it is strongly oriented towards the protection of the users’ privacy. Finally, its implementation requires minimal efforts since it employs the existing infrastructures and devices, thus giving life to a new and wide data environment, exploitable in many domains, such as e-health, smart cities, and smart mobility.

## 1. Introduction

Our everyday activities produce an ever increasing amount of information, as each of them is accompanied by a large number of devices aimed at supporting us (e.g., smartbands, credit cards, home automation devices, and so on). Indeed, several authoritative studies indicate that in 2025 the number of *smartphones* will reach about 7.5 billion units, and the number of *IoT* devices in the same year will be 16.44 billion (https://www.statista.com/statistics/1183457/iot-connected-devices-worldwide/statista.com) (accessed on 1 September 2021). This scenario has been further revolutionized with the advent of cryptocurrencies, in particular *Bitcoin* [1], which have traced a new paradigm for the decentralization of services. A synergistic combination of *security* and *anonymity* is the basis of their success, since this paradigm allows the users to exchange money without involving trusted authorities as intermediates. The strategy behind this revolutionary way to operate is mainly based on the exploitation of digital signature schemes, to implement a fully-decentralized and immutable public ledger where all the transactions are recorded. Such a ledger is known as the *blockchain* and involves a distributed consensus protocol operating in a peer-to-peer network [2].

The paradigm proposed in this work, here called *Internet of Entities* (*IoE*), revolves around the *wireless-based* ecosystem. Some existing devices (hereinafter referred to as *trackers*) are used to track the activity of other devices strictly associated with people or things (hereinafter referred to as *entities*), registering the collected information in a permanent way by leveraging the features offered by a blockchain-based *distributed ledger*.

**Figure 1:**
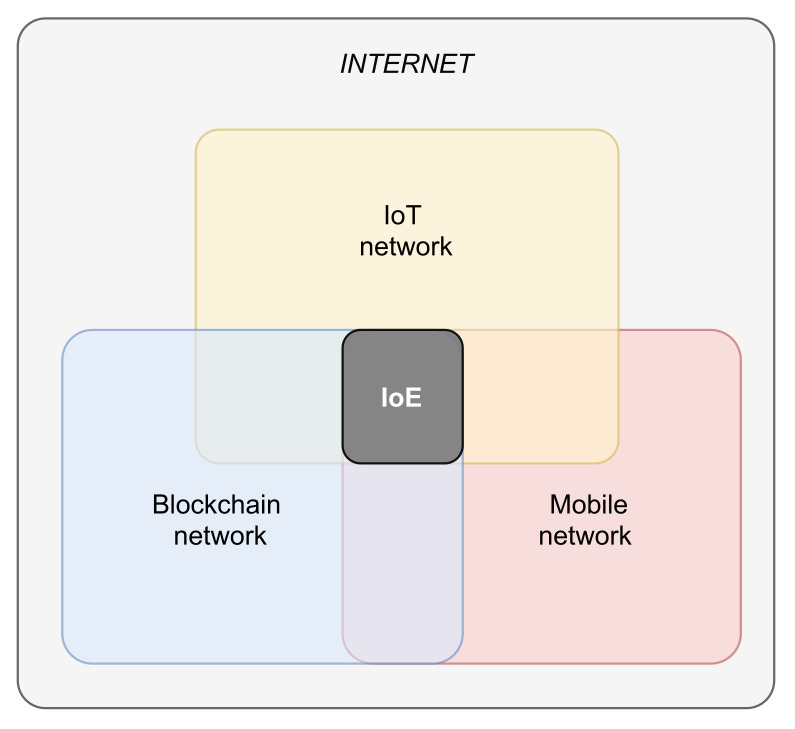
IoE placement.

Figure 1 shows the placement of the proposed *IoE* paradigm with respect to the existing network technologies. Notably, it is designed to require a minimum set of additional functionalities for the existing devices (e.g., smart objects, smartphones, etc). In short, we aim to combine a few *entity* data (i.e., *unique identifier* and sensors data) with a few *tracker* data (e.g., *timestamp*, *geographic location*, *sensors data*, etc.) and store them in a blockchain-based distributed ledger, to implement reliable tracking/localization services. Hence, for devices such as smartphones and tablets, the required operations can be performed in a pretty transparent way by installing a simple application, while for the *IoT* devices, they only require a software update.

Furthermore, the proposed paradigm ensures the privacy of users, since only the entity owner is able to associate its unique identifier to the related data registered by the *trackers* on the remote ledger. In this sense, the possibility of involving one or more *neighbor entities*, detected by the *tracker* near the *entity*, over a certain time window, leads to more granular and accurate tracing results, without violating the anonymity of the involved users.

Although tailored to the scope of *localization*, this approach can be also exploited in other areas, such as *e-health* and *smart cities*. In the first case, the sensor data available for *trackers* (e.g., humidity, temperature, light level, altitude, position, etc.) together with the sensors available on the *entities* (e.g., heart rate, blood pressure, etc.) can be exploited to evaluate the health status of an *entity*. Moreover, one of the advantages related to such a configuration is the capability to monitor the interactions (even those latent) between the *entities* and the environment. Similarly, in the context of smart cities, such an approach helps to highlight latent aspects related to the interaction between users, otherwise difficult to identify. In this sense, it offers a twofold advantage: it is able to discover latent characteristics of the involved *entities*, and it is able to group them on the basis of certain criteria, safeguarding their privacy.

In the light of the previous observations, we list below the primary scientific contributions of the *IoE* paradigm proposed in this work:

(i) Definition of the *entities* and *trackers* concept as elements able to exchange their role when they operate within a specific *wireless-based* environment;
(ii) Formalization of data exchange methods between *entities* and *trackers*, and *trackers* and *blockchain-based distributed ledgers*, in terms of both device identification and communication protocols;
(iii) Formalization of the data structures involved in the *entities* and *trackers*, and *trackers* and *blockchain-based distributed ledger* communications.

In addition, since this work represents an extension of our previous one [3], we extended the aforementioned contributions by adding the following ones:

(i) A comprehensive discussion of the context and the scientific background in which the paradigm proposed in the present work is placed;
(ii) An extended formalization of the notions of *entities* and *trackers*, able to interchange their roles, which operate within the *wireless-based* environment to provide secure *localization services*;
(iii) A timely definition of the interaction model and communication protocols between *entities*, *trackers* and the blockchain-based *distributed ledger*, with the goal of reliably and permanently recording tracking information in a verifiable fashion and respecting their privacy;
(iv) The identification of specific heuristics and strategies, to reconstruct the *entities* movements by means of the history of positions stored on the distributed ledger;
(v) A series of experiments aimed to verify and evaluate the practical feasibility of the proposed *IoE* paradigm in a real-world scenario;
(vi) An in-depth analysis and coverage of the potential usage applications, future developments and implementation directions of such a paradigm.

We also notice that the adopted terminology (i.e., *entities*, *trackers*, and their operative environment) is purely functional to the description, since it better exemplifies the features of these components in the context of the proposed paradigm, therefore it can be different from the canonical definitions of these elements (e.g., client, proxy, etc.).

The paper is organized as it follows: Section 2 provides a detailed overview of the background and related work; Section 3 illustrates the adopted formal notation; Section 4 describes the approach formulation of the proposed paradigm;  Section 5 reports the results of the experiments performed in order to verify and evaluate the practical feasibility of the proposed paradigm in a real-world scenario; Section 6 analyzes the potential applications, limitations and future directions of *IoE*; Section 7 closes the paper with some concluding remarks.

## 2. Background and Related Work

This section aims to introduce the most important concepts exploited in this work. It starts by offering an overview of the *Mobile Network* and *Internet of Thing* environments, then delving into the notion of *blockchain* and its applications. Finally, it concludes with some considerations on the security aspects related to the aforementioned scenarios.  

### 2.1. Mobile Environment

The *mobile* (or *cellular*) environment is a radio network distributed over land areas (defined *cells*), where each cell is served by at least one base station (i.e., a fixed-location transceiver) [4]. This configuration divides the radio network into overlapping geographic areas, producing a mesh of hexagonal *cells* served by a base station (bs) placed in the center of the area. The cell overlapping grants a continuous radio coverage of the connected mobile devices since each base station operates like an hub, retransmitting a mobile device’s radio signal to another mobile device, and adopting different frequencies to avoid interferences. Moreover, the base stations grant the connection between mobile devices when they move between cells.

The proposed *IoE* paradigm can exploit such an environment since the radio coverage allows data exchange between *entities* and *trackers* and between *trackers* and distributed ledgers.

### 2.2. IoT Environment

The Internet of Things (IoT) environment involves a huge number of devices with access to the Internet, and their number is constantly growing day by day. This is mainly given by the concordance of two factors: the low cost of the devices and the significant number of possible practical applications. Canonical devices such as computers, smartphones, and laptops are combined with other devices such as wearable ones, IP cameras, and many others, generating a novel ecosystem where each element can communicate without any geographical limitation.

**Figure 2:**
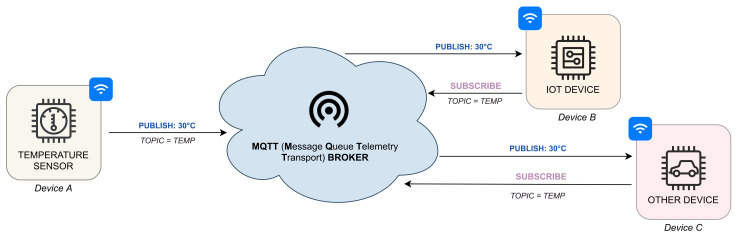
IoT communication paradigm.

This new communication paradigm, where each device can communicate with another one, is simplified in Figure 2, which shows how it is possible to exchange information between geographically distant devices using the *Message Queue Telemetry Transport* (MQTT) protocol. In more detail, this process is performed by using two operations, publish and subscript. First, through the MQTT protocol, a device publishes data on a server (conventionally called *Broker*). Then, other devices can receive the data by subscribing to a *topic* (a channel mechanism that allows us selective intercommunication between devices) where they are stored.

**Internet of Everything**. The rise of the *IoT* model and, more generally, the development of *wireless-based* technologies has contributed to the definition of a model called the *Internet of Everything* [5], further pushed forward by the advent of *smartwatches*, *e-health devices*, *smart vehicles*, to name a few. With respect to the Internet of Things, the Internet of Everything model is used to refer to the intelligent interconnection of *data*, *processes*, *things*, and *people*, a scenario that involves billions of objects connected over public or private networks by using different protocols (*standard* or *proprietary*), which can detect the environment around them (*sensors*) or can interact with it (*actuators*).

**Identity of Things**. The *Identity of Things* represents a concept mainly related to the *Internet of Things* environment. It refers to assigning a unique identifier to all objects operating in such an environment to allow their real-time interaction with people and other objects (*things*). A centered and quite recent example of the scenario mentioned above is that of *autonomous vehicles* [6], where the concept of unique identification becomes day after day even more crucial [7]. The identifier can be created by using information that uniquely characterizes the *IoT* device, such as the manufacturer, the serial number, and so on. Alternatively, the identifier can be assigned to the *IoT* device by using a centralized or decentralized assignation remote service, manually or automatically. Some possible approaches able to perform this operation are presented in Section 4.1.

### 2.3. Blockchain Environment

The *blockchain* data structure has been introduced in the context of cryptocurrencies like *Bitcoin* [2,8] and, later, *Ethereum* [9], as a shared and transparent *distributed ledger* to store and trace financial transactions. It can be imagined as an ever-growing chain of blocks, where each block stores a set of transactions and contains a cryptographic signature of its predecessor, thus making it computationally hard to alter or remove the older blocks. However, its functionality can also be exploited in non-financial contexts, in all the cases where an application needs to ensure trust services. In other terms, it can be employed to define the underlying trust level of an application. For example, its ability to verify identity through a reliable authentication process [10] is nowadays exploited in the context of heterogeneous environments, such as, for instance, those related to *e-health* [11], *smart cities* [12], and *IoT* [13] applications.

**Figure 3:**
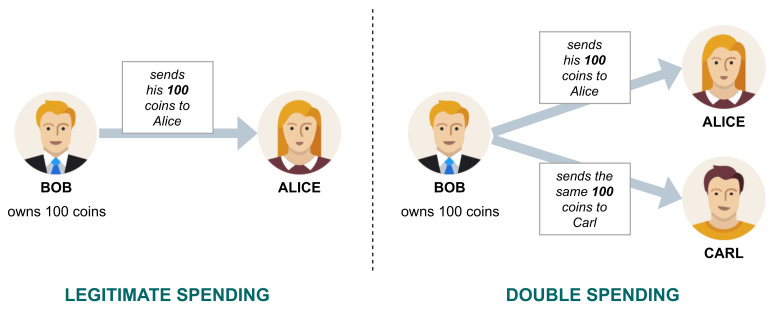
Double spending issue.

**Double Spending**. The main peculiarity of the blockchain is that it can guarantee (or strongly limit), in a decentralized manner, the presence of *double spendings*. These issues arise from the absence of a central intermediary in the decentralized context of the blockchain. Roughly, let us assume that an *entity Bob* owns 100 and *binds* them in a transaction to pay another *entity Alice* for a certain service/good. A double spending occurs if *Bob*, illegitimately, binds the same 100 in a second transaction, to pay another *entity* (e.g., *Carl*) for a different service/good. Since there is no central intermediary (e.g., a bank), it may be challenging to determine that one of the two transactions was invalid. This problem, graphically summarized in Figure 3, has been faced by adopting a distributed timestamp mechanism to determine which transactions should be accepted and rejected. More specifically, common blockchain technologies (like, for example, Bitcoin) exploits a *hash-chain* approach to address this task [11]. To clarify this point, suppose that a block $B_{0}$ stores the transaction from *Bob* to *Alice* and a subsequent block $B_{1}$ stores the transaction from *Bob* to *Carl*: through the hash-chain mechanism, each participant can verify that $B_{0}$ is older than $B_{1}$ and thus reject the double spending transaction from *Bob* to *Carl*.

**Consensus Mechanism**. The *consensus mechanism* stands at the base of the blockchain paradigm: it allows the system to append new blocks to the blockchain, by preserving the hash-chain and avoiding double spendings and *forks*. The literature defines as *validators* the blockchain nodes that participate in the consensus process. More specifically, the most famous consensus mechanism, first introduced by Bitcoin [2], is the so-called *Proof-of-Work*: it assumes that each validator (known as *miner* in the context of this cryptocurrency) employs some large *computational power* to solve a cryptographic puzzle, for earning the right to append the current block to the blockchain. It is is aimed to protect the system against alterations and other fraudulent activities, since it usually requires a very high computation load, which involves resources that are generally not available for a single user or a small group of users. Nowadays, the literature offers other consensus mechanisms to effectively replace the PoW, such as, for instance, the *Proof-of-Stake* (*PoS*) [14,15,16].

**Figure 4:**
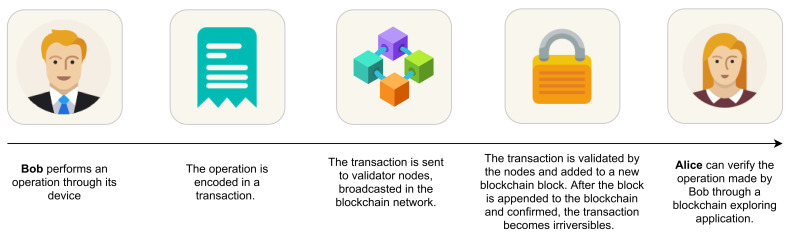
The blockchain as a distributed ledger.

**Blockchain as a Distributed Ledger**. The core of functionality of the blockchain is the possibility to exploit it as a *Distributed Ledger Technology* (DLT). The insertion and validation of operations carried out by leveraging on the blockchain as a distributed ledger has been exemplified in Figure 4. There exist mainly two types of distributed ledgers: *unpermissioned* and *permissioned*. Well-known examples of unpermissioned ledgers are the *Bitcoin* and *Ethereum* environments, designed to be open and uncontrolled. This is in contrast with the permissioned ones, where only granted users can append new blocks to the ledger. In any case, using the blockchain as a distributed ledger model implies to store a very large amount of data. with a constant and continuous increasing in its size, thus raising a crucial *scalability issue* that must be effectively faced in the future [17].

**Blockchain and IoT Integration**. From a general point of view, the blockchain fits well in all the applications where there is the need to *identify entities* (e.g., people, vehicles, documents). For instance, [18] uses blockchain to get a verifiable identity through a reliable authentication process; [19] introduces *blockchain-based* intelligent transportation systems; [20] exploits blockchain to define a public identities ledger in the context of an identity management system; [21] faces the *Value Added Tax* (*VAT*) fraud problem. More specifically, literature discusses scenarios characterized by the integration of blockchain-based infrastructures with *IoT* devices, such as in [22], where the authors identify the following operative modalities:

- *IoT-IoT*: characterized by low latency and a high level of security, since the involved *IoT* devices operate between them for most of the time, by exploiting the  canonical protocols and by limiting the blockchain use for storing only a few information;
- *IoT-Blockchain*: by following this strategy, all the *IoT* information is stored on the blockchain, assuring its immutability and traceability, but increasing the bandwidth consumption and the latency-time;
- *Hybrid Paradigm*: this last strategy combines the two previous ones, performing part of the activities directly between the *IoT* devices, and limiting the interaction with the blockchain to the data storage activity.

For the needs of the proposed *IoE* paradigm, the second and third strategies (i.e., *IoT-Blockchain* and *Hybrid*) are the most suitable. However, by adopting optimized criteria, the best method results in the *Hybrid* one, since it better balances the advantages offered by the *IoT-IoT* and *IoT-Blockchain* strategies.

### 2.4. Security Aspects

Many works in the literature [23] evidence that *wireless-based* technologies evolution did not keep up with the security one. It means that a series of problems that affect security in a broad sense jeopardize the significant opportunities offered by these new technologies. Some cases in point are fraud related to the e-commerce infrastructure [24], where several approaches, proactive and retroactive, have been experimented in order to face such problems [25], as well as the ever-increasing number of identity theft [26] or, even more simply, the countless frauds made by exploiting the people’s trust [27], often by recurring to social engineering techniques [28].

Furthermore, in the mobile network context, we can observe similar problems because the smart devices that operate in this environment inherit the security risks that characterize the Internet-based devices (e.g., desktop computers, laptops, and so on), such as the ones above. In addition, there are a series of more specific risks related to this context [29], such as, for instance, those related to *bot-net-based* attacks [30], or those that jeopardize user privacy [31].

Security issues also flank the potential advantages of blockchain-based technologies, where criminals try to exploit them fraudulently. Moreover, surveillance authorities can not easily detect such criminal activities [32]. Another example of a security issue related to the blockchain concerns the Proof-of-Work consensus.

As a group of people can operate jointly (i.e., forming a *mining pool*) to reduce time variance in the mining of new blocks, if they achieved a grouped computational power that is at least 51% of the total of the network, they can gain a full control of the blockchain, breaking down its decentralization [33,34]. This attack has been theorized in the literature as the *majority attack* [2], but never occurred in real use.

### 2.5. Localization Data Encoding

Blockchains have various ways to encode and store timestamps, locations, and further metadata (i.e., data not related to financial operations). These techniques vary depending on the target blockchain. Indeed, since Bitcoin’s birth, several applications have exploited different fields of its protocol to append metadata for various use cases, including tracking objects [35]. The most common technique in the Bitcoin blockchain, which also applies to Litecoin, exploits the OP_RETURN field [36]: applications append a string of data encoded by following a personal protocol. After a few years, Ethereum [9] arose, providing the (currently) most widespread technique for encoding data and performing computations on blockchains: the smart contracts.

More recently, permissioned blockchains like Hyperledger Fabric sprung up [37]. They still use smart contracts. However, they differ from previous (permissionless) blockchains because they do not have a public network, releasing users from the disadvantages of a public fee but requiring them to configure and maintain a custom network. For this reason, the cost of a transaction in a permissioned system may highly vary, and it is not possible for us to estimate it. Accordingly, we did not include this kind of blockchain in our analysis, although we remark that Hyperledger Fabric is widely used for tracking positions and the life cycle of objects [38], to emphasize the generality of our paradigm.  

### 2.6. Related Work

The literature presents several approaches that face similar tasks to those proposed in this manuscript. Although the idea behind almost all of these works covers specific application areas, it can not be considered a paradigm extensible across varied domains, even very different ones, as in our case. For instance, in [39] the authors consider the blockchain technology as a business strategy, proposing and discussing its application in the fresh food delivery area, also proposing some considerations about the use of this technology in reducing the logistics costs. Similarly, in [40], the authors propose a blockchain-based framework to automate business flows in tracking supply chain processes. Other works exploit the capabilities of blockchain, such as in [41], where the advantages of the blockchain are combined with machine learning algorithms in the education field, or in [42], where instead the 5G cellular network is combined with the blockchain technology in order to provide reliable communication and enhanced information security.

Practically, what characterizes and differentiates the proposed *IoE* paradigm from the other works in the literature is that we define a common approach potentially exploitable on a wide variety of application domains rather than on a specific one.

## 3. Formal Notation

We use the term *entity* to indicate a device designed to operate in an *IoE* environment, strictly associated with a person or thing. We use the term *tracker* to indicate a generic (new or already existing) device that operates in a *wireless-based* environment, which is aimed to interact with the *entities*. Given the above considerations, we introduce the following formal notation:

(i) We denote as $E=\{e_{1},e_{2},\ldots ,e_{M}\}$ a set of *entities*, and we use E(e) to indicate such information related to an *entitye*;;
(ii) We denote as $E_{\tau }=\left\{e_{1},e_{2},\ldots ,e_{N}\right\}$ the *entities* in *E* detected by a *tracker* device within τ seconds after detecting an *entity* (then $E_{\tau }\subseteq E$), and we use $E_{\tau }\left(e\right)$ to indicate such information related to an *entitye*;
(iii) We denote as $L=\{l_{1},l_{2},\ldots ,l_{O}\}$ a set of geographic locations, with l=latitude, longitude, and we use l(e) to indicate such information related to an *entitye* when a *tracker* device detects it;
(iv) We denote as $T=\{t_{1},t_{2},\ldots ,t_{P}\}$ a set of *timestamps*, with t={yyyy−mm−dd−hh−mm−ss}, and we use t(e) to indicate the *timestamp* related to detecting an *entitye* by a *tracker* device;
(v) We denote as $I=\{i_{1},i_{2},\ldots ,i_{Q}\}$ a set of GUIDs (i.e. *Globally Unique IDentifiers*, whose structure is formally defined in the *RFC-4122* and explained in Section 4.1), using the notation i(e) to indicate the GUID associated with an *entitye*, and the notation i(tracker) to indicate the GUID associated with a *tracker* device;
(vi) We denote as $P=\{p_{1},p_{2},\ldots ,p_{W}\}$ a *payload*, with p={key,value}, and we use P(e) to indicate a *payload* related to an *entitye*;
(vii) We denote as $R=\{r_{1},r_{2},\ldots ,r_{Y}\}$ a set of registration made on a *blockchain-based* distributed ledger, with $r=\{i\left(e\right),E_{\tau }\left(e\right),l\left(e\right),t\left(e\right),P\left(e\right)\}$, and we use r(e) and R(e) to indicate, respectively, a registration related to an *entitye* and all the registrations associated with that *entity*.

## 4. Approach Formulation

This section describes the four steps required to define the proposed *IoE* paradigm; we summarize them in the following:

(i) **Elements Definition**: it introduces the concept of *entity* and *tracker* in the *IoE* environment, as well as the method to assign them a *Globally Unique Identifier*, outlining some possible operative scenarios;
(ii) **Elements Detection**: the detection process of an *entity* device is here described, from the *detection-time* by a *tracker* device to the *recording-time* of the collected data on a *blockchain-based distributed ledger*, focusing on the characteristics of the state-of-the-art wireless technologies able to perform these activities;
(iii) **Elements Communication**: it formalizes the data structures and the software procedures able to merge the information related to the involved *entity* and *tracker* devices, generating the *data structure* that represents the information to store on the *blockchain-based distributed ledger*;
(iv) **Elements Localization**: extensively, it describes the activities made to trace an *entity*, introducing some baseline strategies and a series of localization rules aimed to exploit the available information on the *blockchain*, directly or indirectly.

### 4.1. Elements Definition

*Entities* can be either persons or objects, like *vehicles* or *goods*. Each *entity* is always associated with a *Globally Unique Identifier* (*GUID*).

Conversely, *trackers* usually are generic devices able to detect *entity* devices, capturing their *GUIDs* and sensors data, and performing a registration into a blockchain-based distributed ledger. Such a registration (i.e., the set *r*) is defined by joining *entity* and *tracker* data, according to the formal notation defined in Section 3.

The unique identifier of the *tracker* devices could be already available (e.g., *MAC-address*, *IP-address*, etc.), while that of the new *entity* devices placed in the *IoE* environment needs to be defined and assigned. There exist several ways for generating unique identifiers [43], but the two most common methods use: (*i*) *serial numbers* created by following an incremental or sequential criterion; (*ii*) *random numbers* generated by using a range of numbers enough larger to classify the expected number of objects. In the proposed approach, we perform this operation using one of the most effective methods: the *Globally Unique Identifier*.

**Globally Unique Identifier**: The *Globally Unique Identifier* (*GUID*), also known as *Universally Unique Identifier* (*UUID*), is a 128-bit integer number commonly used to identify resources uniquely [44]. In particular, [44] also provides a formal definition of *GUID* string and algorithms able to generate it: for instance, *f81d4fae-7dec-11d0-a765-00a0c91e6bf6* represents an example of *GUID* string.

Through the application of the *birthday paradox* [45], we can obtain a mathematical demonstration of the *GUID* robustness in terms of hash collision probability. By following this mathematical approach, considering that a *GUID* is a *128-bit* long number, we can identify a  quadrillion *entities*  (i.e., $10^{15}$) before we have a one in a billion possibility to get a collision.

Some considerations can be made about the policies to adopt in order to assign the *GUID* to each *entity* device that operates into the *IoE* environment, assuring that this information remains stable along the time. This is because the *IoE* tracing mechanism uses such information and a variation of it (i.e., the device *GUID*) during the life of an *entity* device leads towards inconsistent data.

Some solutions involve a centralized *GUID* distribution, such as in [46], offered as service to the users by following a free or paid modality, or an autonomous generation of this information made directly by the users [44]. It is appropriate to reserve part of the *GUID* information to distinguish the *IoE* devices from the other devices operating in the wireless-based environment.

**Operative Scenarios**: About the hardware to use in the *IoE* environment for allowing the *entity* devices to interact with the *tracker* ones, we can outline several scenarios:

(i) The *entity* device has limited or absent hardware resources (e.g., *CPU*, *memory*, etc.), then it performs the identification process by exploiting passive technologies like *RFID* (Radio-Frequency IDentification). In this first scenario, the *tracker* device must be able to manage the identification process adopted by the *entity*;
(ii) The *entity* device has hardware resources to adopt active technologies for the identification process (e.g., *6LoWPAN* and *ZigBee*, both defined by the *technical standard IEEE 802.15.4*). This is the most common scenario, where the *entity* device uses canonical wireless technologies and the *tracker* device does not need any additional capability to interact with it;
(iii) The *entity* device can perform processes that require considerable hardware/software resources. Such a scenario allows us to move on the *IoE*-side some processes usually performed on the tracker-side, and it also allows the *IoE* device to handle complex processes related to its sensors.

The scenario we consider in this paper is the second one, where the *IoE* device has enough hardware/software resources that allow it to use active technologies for its identification because it enables us to implement the *IoE* immediately and transparently, postponing the other scenarios to possible future implementations.

### 4.2. Elements Detection

**Figure 5:**
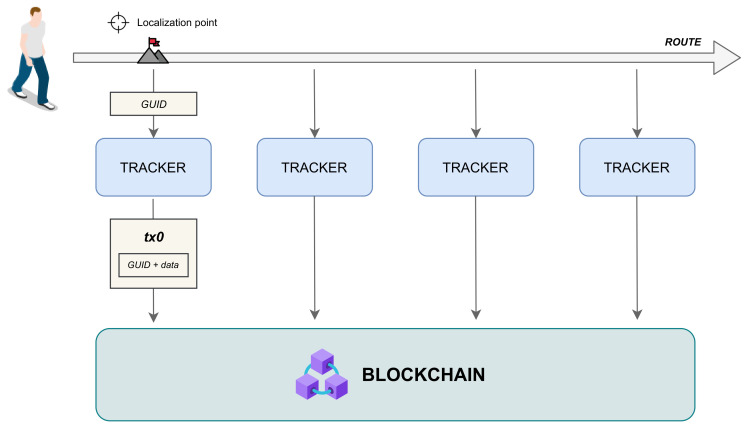
Working model of the Internet-of-Entities.

As shown in the high-level working model of Figure 5, when an *entity* *e* enters within the coverage area of a *tracker* device, such a device detects its identifier *i* (i.e., the *GUID*, as formalized in Section 3), and it creates and submits a registration *r* on a *blockchain-based distributed ledger*.

Figure 5 indicates the detection time of an *entity* *e* as *data capture*, and it coincides with the *timestampt*, which represents the point in the space where a *tracker* device detects the *entity* and submits the information *r* to the *blockchain-based distributed ledger*.

All the above operations are managed using specific data structures, whose possible implementation has been proposed in Section 4.3.

**Wireless Technologies**: Regarding the technology to use for broadcasting the *entity GUID*, the literature offers several technologies and protocols to perform this operation [47]. Some examples of them are: *Internet Protocol Version 6 over Low-Power Wireless Personal Area Networks* (*6LoWPAN*), *Bluetooth Low Energy* (*BLE*), *Z-Wave*, *ZigBee*, *Near Field Communication* (*NFC*), *Radio Frequency IDentification* (*RFID*), *SigFox*, and *2G/3G*. *SigFox* and *2G/3G* are classified as *Low-Power Wide Area Network* (*LPWAN*) protocols, while the other ones as *Short-range Wireless* protocols.

Table 1 summarizes their characteristics, where the reported ranges (i.e., *frequency range* and *operative range*) indicate only the lowest and the highest supported value (e.g., if the protocol supports 125 KHz, 13.56 MHz, and 860 MHz, we report 125 KHz ÷ 860 MHz).

The protocol choice should take into account the *entity* type. For example, in the case of a *person*, such a choice should be oriented toward protocols able to ensure a low-power consumption and a mid/short operative range. While in the case of *objects* (e.g., a vehicle), the choice could be instead oriented toward protocols characterized by a long operative range and a mid/high power consumption.

However, the above considerations are strongly related to the context of a custom *IoE* device: when it is a standard device such as, for instance, a smartphone or a tablet, the choice of the wireless protocols is driven by those supported by the operating system (e.g., *802.11 b/g/n* [48] and *Bluetooth Low Energy* (*BLE*) [49] protocols).

### 4.3. Elements Communication

**Figure 6:**
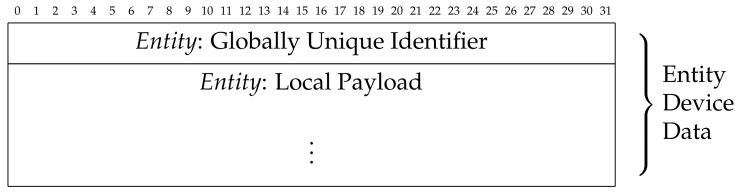
*Entity-side* data structure.

**Figure 7:**
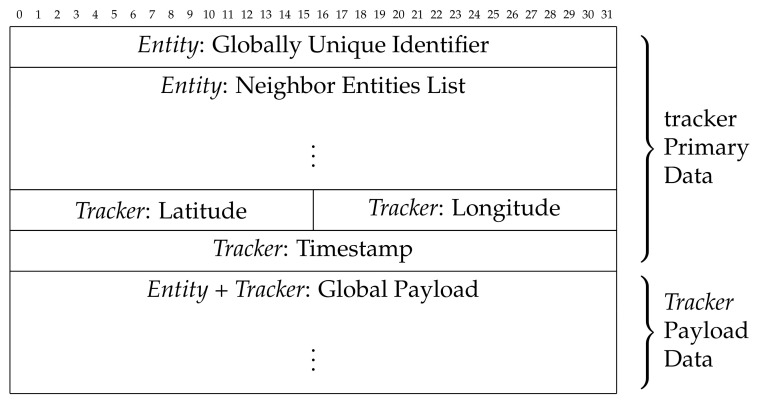
*Tracker-side* data structure.

The communication between an *entity* *e* and a *tracker* device can be performed by adopting elementary data structures, whose possible formalization is proposed in Figure 6 and Figure 7.

They refer, respectively, to the data structure used to transmit data from an *entity* device to a *tracker* device (i.e., *entity-side*) and to the data structure used to transmit the registration data from a *tracker* device to the *blockchain-based distributed ledger* (i.e., *tracker-side*).

About the *Entity-side* data structure, the *GUID* information, 128-bit long, is stored by using five groups of hexadecimal digits, with the following sizes: eight hexadecimal digits, four hexadecimal digits, four hexadecimal digits, four hexadecimal digits, and 12 hexadecimal digits.

The registration data *r* are defined by merging a series of identification data (*Tracker Primary Data*) with the sensors data related both to the *entity* and *tracker* devices activity (*Tracker Payload Data*). In some contexts, the *Payload Data* could be partially (only the *entity* or *tracker* sensors data) or completely absent (no sensors data), and, in these cases, the *entity* information will be the *GUID*, the *location*, and the *timestamp*.

The hardware/software process performed on the *entity-side* is limited to broadcast its data (*GUID* and *local payload*) at regular intervals using the wireless functionality. Regarding the *tracker-side* hardware/software process, when there are no other priority tasks active, the *tracker* device operates a listening activity to detect *entities* in its wireless coverage area, sending the collected *entity* and *tracker* data to the *blockchain-based distributed ledger*.

Notably, in the data structures, we classify the *payload* based on the data it refers to, using the term *local* to indicate that generated by the *entity* device and *global* to indicate that generated by the *tracker* device, which also includes the *local payload*.

The *data anonymity* and *data immutability* offered by a *blockchain-based distributed ledger*, joined with the low cost of the devices needed for the data transmission and the wireless coverage provided by the ever-increasing number of *wireless-based* devices, give life to a robust environment on which the proposed *IoE* paradigm is based.

The data that we need to store on the *blockchain-based distributed ledger* is that described in Section 3: the first field *i* contains the *Globally Unique Identifier* of the *IoEentity*; the field $E_{\tau }$ contains, when it is applicable, a list of *Globally Unique Identifiers* related to the other *entities* captured together with the *entitye* in a defined temporal frame τ; the *l* field contains the geographic position (i.e., *latitude* and *longitude*) of the e-health device that detected the *entitye*; the field *t* reports when the *data capture* event occurred, in the format *yyyy-mm-dd-hh-mm-ss*; the last field *P* contains a series of values in the format *key,value* which refer to the sensors data of the *entity* device (*local payload*) and the sensors data of the e-health device (*global payload*).

**Software Procedures**: The software to perform the *entity-tracker* and *tracker-ledger* communications can be an update, in case of *IoE* and *custom devices*, or an application (*app*), in most other cases (i.e., *smartphones*, *tablets*, and similar devices). It has to fulfill the *IoE* paradigm needs, from the *entity detection* to the *data registration*, by performing the following operations:

1. *entity-side*: it provides to broadcast the device *GUID* along with the *payload* (i.e., local sensors data) by using the built-in wireless device functionality;
2. *tracker-side*: it performs a listening activity aimed to detect and recognize (distinguishing them from the other devices through the mechanism adopted in the implementation phase, for instance, a specific *GUID* preamble) *entities* within its wireless coverage area;
3. *tracker-side*: it appends the e-health device data (i.e., *primary* and *payload* data) with the data transmitted by the *entity* device (i.e., *GUID* and *payload*), building a data packet suitable for registration on the *blockchain-based distributed ledger*;
4. *tracker-side*: it submits the defined data packet on the *blockchain-based distributed ledger* to perform an immutable registration of the *entity* device activity;
5. *tracker-side*: it waits to receive from the *blockchain-based distributed ledger* the registration acknowledge of the submitted packet; otherwise, it repeats the submission.

A series of custom *data dashboards* (i.e., management tools able to display, track and analyze information) can also be designed to manage all the processes involved in the *IoE* paradigm, which is related to the constant tracking of the *entities*.

### 4.4. Elements Localization

**Figure 8:**
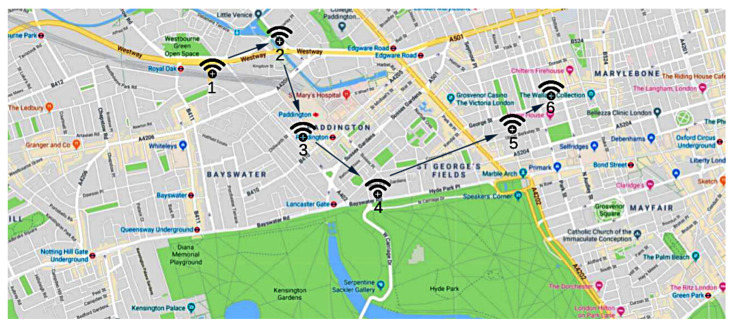
IoE direct tracing.

When we need to investigate an *entity* *e*, first we get all needed data related to it by performing a *data gathering* process, such as that reported in Algorithm 1, then we can manage such data through different strategies, such as the baseline ones described below:

1. **Direct Tracing**: by following this strategy, the movements of an *entitye*, from its first introduction in the *IoE* environment, are traced by using the information l(e) and t(e) in r(e),∀r(e)∈R(e), according to the formalization given in Section 3.
  Figure 8 shows this process, and presents six detection points *l* of an *entitye*, chronologically numbered by using the *timestamp* information *t*. In more detail, we first query the *blockchain-based distributed ledger* to extract all the registrations R(e), and then we number each location l(e)∈r(e),∀r(e)∈R(e) (i.e., *latitude* and *longitude*) along the chronological sequence given by the *timestamp* information t(e)∈r(e).
  More formally, given a series of *entity* locations l(e)∈L, we introduce a *Trace Location Set*$\omega =\{l_{1},l_{2},\ldots ,l_{Z}\}$ to store all the locations l(e)∈L in the chronological order determined by the *timestamp* information t(e)∈T, as formalized in Equation (1).
  (1) $$\begin{array}{c} \omega \leftarrow \;l(e)\;\;|\;\;\forall \;\;l(e)\;\;\mathrm{in}\;\;L\;\;\\ \mathrm{with}\;\;l_{1}<l_{2}<\ldots <l_{Z}\;\;\wedge \;\;l\in \omega \end{array}$$
  ** Algorithm 1** Blockchain-based distributed ledger data gathering.**Require:***e* = Entity, *R* = Blockchain-based distributed ledger registrations
**Ensure:**
$\hat{R}$ = Registrations related to entity *e*1:**procedure** getEntityRegistrations(*e*, *R*)2:    **for each** *r* **in** *R* **do**3:        i←getEntityGUID(r)4:        **if** $i\left(e\right)==\hat{e}$ **then**5:           $\hat{R}\leftarrow r\left(e\right)$6:        **end if**7:    **end for**8:    **return** $\hat{R}$9:**end procedure**

**Figure 9:**
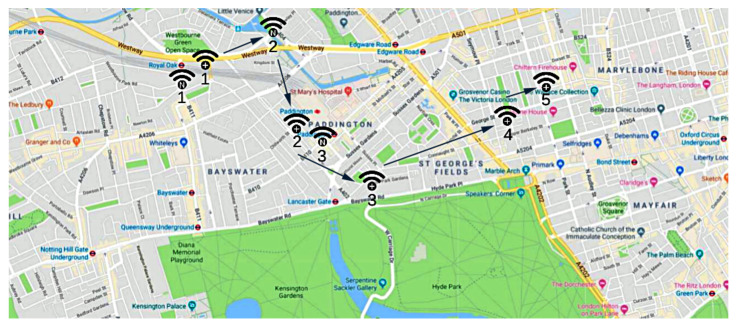
IoE interpolate tracing.

The localization resolution is directly related to the e-health device that has detected the *entity*. We can obtain a *high-resolution* localization when the e-health device runs a localization service (e.g., *GPS*) an its location is near the detected *entity*. Instead, we obtain a *low-resolution* localization when the localization data are related to another device. For instance, this happens when the e-health device operates in the mobile network but without any active localization service. In this case the location could refer to the mobile network *cell*.
  This is represented in Figure 8 and Figure 9: the *high-resolution* localization coincides with the *entity map-point*, while the *low-resolution* localization can be considered any *map-points* within the *grid-square* where the *entity* is placed, which represents the mobile network *cell*.

**Figure 10:**
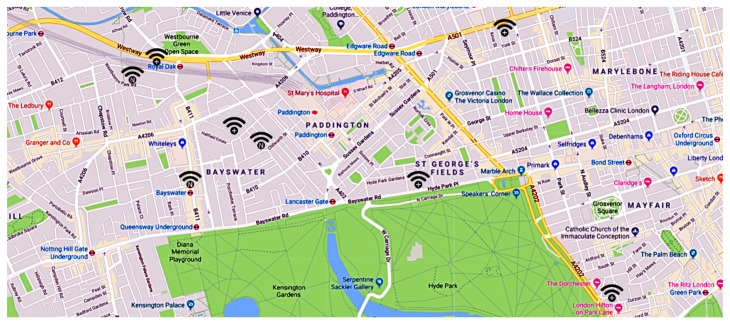
IoE spread tracing.

2. **Interpolate Tracing**: in this strategy, we take into account the information l(e), t(e), and $E_{\tau }\left(e\right)$ in r(e),∀r(e)∈R(e). When applicable, the $E_{\tau }\left(e\right)$ information contains the other *entities* detected by the e-health device within τ seconds from the detection of *e* (the *entity* under analysis), as described in Section 3.
  We exploit the new information to reconstruct the *entity* movements by interpolating the l(e)∈r(e),∀r(e)∈R(e) data with the same data of the *entities* in $E_{\tau }\left(e\right)$ (neighbor *entities*). Figure 9 graphically shows this process, where *+* denotes the *entity* under analysis and *N* a neighbor *entity* in $E_{\tau }\left(e\right)$.
  In the example of *interpolate tracing* shown in Figure 9, we can observe how the first localization of the *entity +* includes a neighbor *N* that we found another time in the third location of the location chronology of *+*. This represents a naive example of *interpolate tracing*, based on the reasonable probability that such a configuration indicates that the neighbor *entity* is somehow related to the primary *entity* under analysis, especially when this pattern repeats over time. In other words, it is very likely that in the second localization of *N*, the *entity +* was also present and that it has not been detected for some reasons such as, for instance, a temporary e-health device overload, or because the *entity* device was out of the wireless e-health range. This pattern, repeated over time, could underline interesting connections between *entities*, as well as the last location of a missing *entity*.
  More formally, given the *Trace Location Set* $\omega =\{l_{1},l_{2},\ldots ,l_{Z}\}$ previously defined and given a series of *entity* locations $L\left(e\right)=\left\{l_{1},l_{2},\ldots ,l_{O}\right\}$, at each location l∈L(e) (with O≥3) we extract from the set $E_{\tau }\left(e\right)$ a subset of valuable neighbor *entities* by following the criterion in Equation (2).
  (2) $$\begin{array}{c} \omega \leftarrow \;l\left(e\right)\;\;|\;\;\mathrm{if}\;\;e\;\;\mathrm{in}\;\;E_{t}\left(l_{o-1}\right)\;\;\wedge \;\;e\;\;\mathrm{in}\;\;E_{t}\left(l_{o+1}\right),\;\;\forall \;\;e\;\;\mathrm{in}\;\;E_{\tau }\\ \mathrm{with}\;\;l_{1}<l_{2}<\ldots <l_{Z}\;\;\wedge \;\;l\in \omega \end{array}$$
  We can generalize the previous criterion by varying the distance between the step $E_{\tau }\left(e\right)$ (i.e., where we extract the valuable neighbor *entities* from E(e)) and the previous and next step that we take into account. Denoting as α such a distance (i.e., the number of considered locations), we can re-formalize the former criterion as shown in Equation (3).
  (3) $$\begin{array}{c} \omega \leftarrow \;l\left(e\right)\;\;|\;\;\mathrm{if}\;\;e\;\;\mathrm{in}\;\;E_{t}\left(l_{o-\alpha }\right)\;\;\wedge \;\;e\;\;\mathrm{in}\;\;E_{t}\left(l_{o+\alpha }\right)\\ \mathrm{with}\;\;l_{1}<l_{2}<\ldots <l_{Z}\;\;\wedge \;\;l\in \omega \end{array}$$
  We underline that we do not infringe the privacy of the involved *neighbor entities* since the *entity* data are collected anonymously into the *blockchain-based distributed ledger*.
3. **Spread Tracing**: this last baseline criterion exploits all the neighbor *entities* in $E_{\tau }$, with $e\neq \hat{e}$ and $|E_{\tau }|\geq 2$, where $\hat{e}$ denotes the *entity* under analysis.
  As valuable neighbor *entities* of $\hat{e}$, we add all the *entities* in their locations *L* have $\hat{e}$ as neighbor *entity*, as shown in Equation (4).
  (4) $$\begin{array}{c} \omega \leftarrow \;l\left(e\right)\;\;|\;\;\mathrm{if}\;\;\hat{e}\;\;\mathrm{in}\;\;E_{\tau }\left(e\right),\;\;\forall \;\;l\left(e\right)\;\;\mathrm{in}\;\;L\\ \mathrm{with}\;\;l_{1}<l_{2}<\ldots <l_{Z}\;\;\wedge \;\;l\in \omega \end{array}$$
  The result can be expressed as the *tracing matrix* Ξ shown in Equation (5), where each row refers to a different valuable *entitye*. In other words, each matrix-row refers to a different valuable *entity* *e*, and it reports the locations *l* where the *entitye* has the *entity*$\hat{e}$ as a neighbor in $E_{\tau }\left(e\right)$.
  (5) $$\Xi \left(e\right)=\left[\begin{array}{cccc} l_{1}, & l_{2}, & \cdots , & l_{O}\\ l_{1}, & l_{2}, & \cdots , & l_{O}\\ \vdots & \vdots & \ddots & \vdots \\ l_{1}, & l_{2}, & \cdots , & l_{O} \end{array}\right]$$
  After ordering the matrix row elements by location and counting how many *entities* $\hat{e}$ are involved in each matrix column, we can evaluate the probability that the *entitye* was in a specific position, although a e-health device has not detected it. Figure 10 graphically shows this criterion, where the *grid-size* (i.e., *square-side*) represents a tolerance value, which we denoted as Δ. This means that all the *entity detections* that occur in the same *grid square* refer to the same *matrix row index* (i.e., Equation (5)).

The grid of Figure 10 represents different information, with respect to that of Figure 8 and Figure 9, since, in this case, it does not represent the mobile network *cells* but the tolerance value Δ. Moreover, all the previous criteria can be combined for defining a more complex strategy based on different localization rules.

As a final remark, we observe that this work is mainly devoted to provide a conceptual framework that aims at exploiting and combining, synergistically, the different potentials of the *IoT*, mobile and blockchain worlds. A massive implementation and testing of concrete use cases in real-world scenarios have not yet been carried out, although they represent the natural future direction of research towards which this work is heading.

## 5. Experimental Findings

In the light of what we said in Section 4, considering that an exhaustive validation of the proposed *IoE* paradigm would require a wide diffusion, as happened with other paradigms (e.g., *IoT*), the experiments carried out and reported in this section are aimed to verify and evaluate the practical feasibility of the proposed *IoE* paradigm in a real-world scenario.

In this context, the implementation of the proposed paradigm has been tested through a prototype based on several *ESP32* boards, equipped with the Zerynth (zerynth.com) (accessed on 1 September 2021) library, and interfacing with the *Ropsten* blockchain (an *Ethereum testnet*). The purpose of the performed experiments has been focused on the following four fundamental aspects that characterize the proposed *IoE* paradigm:

(i) **definition** of *entities* and *trackers*;
(ii) **detection** of the *entities* within the wireless coverage area of the *trackers*;
(iii) **communication** of data made by the *trackers* on a remote *blockchain-based distributed ledger*;
(iv) **localization** of *entities* by applying the baseline strategies formalized in this paper.

### 5.1. Setup of the Experiments

**Figure 11:**
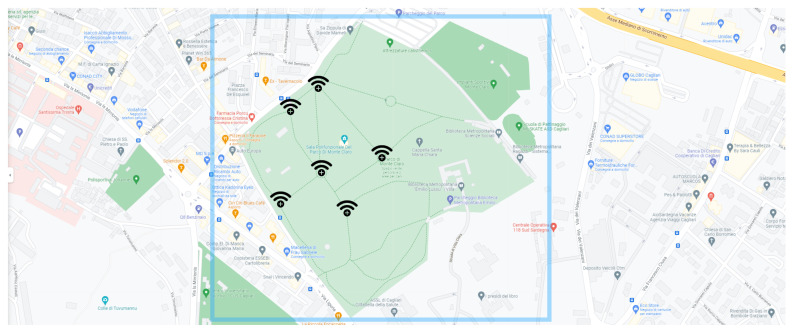
Map of the experiment venue, with the position of each *tracker*.

In order to achieve the set goals, the experiments have been carried out with the aim to simulate, in a restricted context, the real behavior of *entities* and *trackers*, as well as their interaction with the distributed ledger. Specifically, we set up the experiments by first choosing a set of locations $L=\{l_{1},l_{2},\ldots ,l_{j}\}$, where we disposed of *j* *trackers*. Afterwards, we engaged a set of *kentities*$E=\{e_{1},e_{2},\ldots ,e_{k}\}$. We assigned a *Globally Unique Identifier* to each *entity* and *tracker*. Figure 11 shows the main experiment venue, along with *trackers*’ position. For convenience, such a venue were identified as an urban park in Cagliari, Sardinia. Hence, the engaged *entities* were free to move in the venue: whenever one of them met a *tracker*, the communication started and the data were stored in the Ropsten blockchain.

To measure the operational parameters of the paradigm, in each experiment we varied the number of involved *entities* and *trackers*. Moreover, for all of them, we analyzed two different scenarios by evaluating, respectively, the *BLE* technology (Table 2) and the *RFID* one (Table 3). In particular, we use $T_{d}$ to indicate the detection time, i.e., the average time that *trackers* require to detect and perform the handshake with *entities*. Furthermore, we denote as $T_{r}$ the registration time, i.e., the average time required to save a transaction in the *Ropsten* blockchain. Since determining these values for each detection during the experiment was unfeasible, particularly due to the limitations of the boards, they were measured beforehand in a controlled environment, and then kept fixed to calculate the final results. Thus, from the controlled measurements, we obtained, on average, $T_{r}=4.25$ s and $T_{d}=1$ s (regardless of the type of wireless technology considered).

Accordingly, the first goal of our experiments was to determine the *average communication time per tracker* $Avg_{time}$, i.e., the average time spent by each *tracker* to detect and record *entities*, defined as:

(6) $$Avg_{time}=\frac{N_{d}*\left(T_{d}+T_{r}\right)}{N_{t}}$$

where $N_{d}$ and $N_{t}$ indicate, respectively, the total number of detections and the total number of *trackers* of each experiment.

For the sake of completeness, the second goal was to determine the average cost each *tracker* affords to store *all* its transactions in the blockchain. Since the experiments were conducted by means of the Ropsten blockchain, an Ethereum testnet whose transaction cost is free of charge, we simulated the actual cost by considering three different and widely distributed public blockchains, specifically *Ethereum* (as it is already available for use by the prototype), *Litecoin* and *Binance*, that for implementation characteristics are overlapping, but currently more economically advantageous. In light of the above, for each considered blockchain B, we retrieved the average fee cost in *USD* (determined at the time of writing, i.e., *September 2021*); such values, for the three considered blockchains are, respectively, $Fee_{ETH}=7.4$, $Fee_{LTC}=0.016$, and $Fee_{BNB}=0.27$ USD.

With such ingredients, we finally calculated the *average cost per tracker*, for each blockchain, as:

(7) $$Avg_{cost}=\frac{N_{d}*Fee_{B}}{N_{t}}$$

where $Fee_{B}$ is the specific blockchain transaction fee as listed above.

### 5.2. Results and Discussion

The results of the experiments carried out are reported in Table 2 and Table 3 below. The first column defines an unique identifier of each experiment. The second and third columns show the amount of *entities* and *trackers* involved in each experiment. Similarly, the fourth column indicates the total number of *entities* detections $N_{d}$, which also corresponds to the number of blockchain transactions performed to permanently store these events. Finally, the last three columns show the estimated values of average fee per *tracker*, for the three blockchains considered.

What emerges from the results, albeit in a very limited context, but which is fully reflected in real experience, is that the increase in the number of *trackers*—thus indicating a greater pervasiveness of the paradigm—does not seem to affect the average time and cost per *tracker*, since the burden is better distributed among them. However, we observe that *trackers* with a much higher overall burden will always exist, whenever they are associated with inherently busier real-world locations.

The second interesting finding from this preliminary study is that as the number of *entities* increased, average times and costs per *tracker* increased in an apparently linear fashion, regardless of the number of *trackers*. Again, in a massive usage scenario, busier locations will inevitably be subject to a more pronounced growth trend.

Finally, although not the subject of these experiments, we want to recall that it is possible to reconstruct the path of *entities*, *ex post*, by retrieving their positions history from the blockchain and applying one of the localization strategies presented in Section 4.4, i.e., *Direct Tracing*, *Interpolate Tracing*, or *Spread Tracing*.

## 6. Potential Applications, Limitations and Future Directions

This section discusses some future directions where the proposed IoE paradigm is oriented, and it also makes general considerations about its potential spread.

### 6.1. Secure Payload Storing

The first future direction we suggest is an extension of the *IoE* paradigm that could manage as *payload* large and sensitive sensors data by recurring to external storage services and encryption protocols. Such a problem arises concerning the payload data generated by the e-health device that detect an *entity*. Indeed such data could refer to sensitive information generated by some classes of sensors such as, for instance, *microphones* and *video cameras*, instead of non-sensitive information generated by other classes of sensors (e.g., *temperature sensors*, *humidity sensors*, etc.).

A possible and effective solution able to face this problem exploits the *asymmetric encryption* model [57], which analogously to the canonical encryption mechanism adopted nowadays in several applications (e.g., *Secure Socket Shell*, *Open Pretty Good Privacy*, *Secure Multi-Purpose Internet Mail Extensions*, etc.) is exploited for encrypting the data locally (when the e-health functionalities allow us this operation) or remotely (e.g., in a *distributed database*).

**Figure 12:**
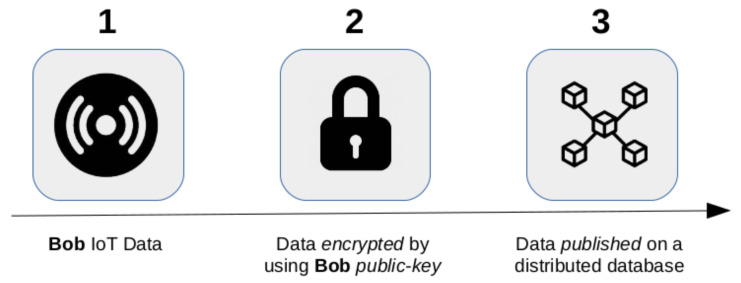
Data encryption process.

**Data Encryption**: The data encryption is performed by using the e-health device *public key*. In this way only it can decrypt the data by using its *private key*, although the involved *entity* has access to that data in encrypted form. Figure 12 summarizes the entire process.

This already happens in the context of the *blockchain* technology, where the private key cryptography mechanism provides a powerful ownership method that fulfills the authentication requirements (i.e., the ownership is *private-key-based*), without the need to share more personal information. In this context, such a mechanism also grants both *privacy* and *ownership*.

When there is the need to investigate an *entity* using such encrypted data (e.g., in case of a criminal event like kidnappings, thefts, etc.), the involved authorities in charge can access data. It is possible to exclude this information using others (e.g., *location*, *timestamp*, etc.) in minor events.

**Data Hashing**: The connection between the encrypted data (stored locally or remotely) and the *entity* is possible using a string generated by a *hash function* as data-name [58]. Such a function is a particular class of *hash* functions largely used in cryptography. Some common examples are: *MD4* [59], *SHA* [60], *TIGER* [61], and *WHIRLPOOL* [62].

In more detail, by adopting a mathematical algorithm is possible to map data (characterized by arbitrary size) to a bit string (characterized by a fixed size). The result is a defined *hash*, and it represents a one-way function that is infeasible to invert. The literature usually refers to the input data as *message* and the output data (i.e., the *hash*) as *message digest* or *digest*.

**Figure 13:**
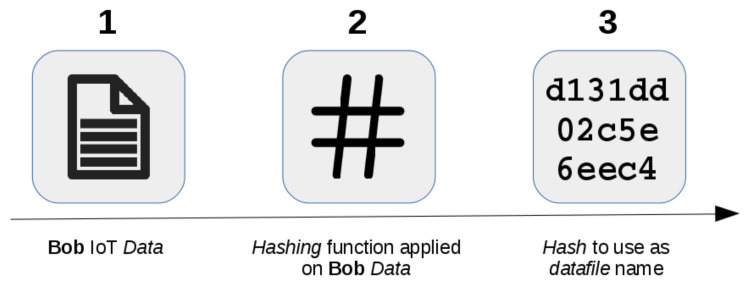
Data hashing process.

A *hash* process shown in Figure 13 makes it possible to validate the file data integrity, detecting all modifications since they change the *hash* output. While an encryption process represents a *two-way function* based on the *encryption* and *decryption* operations, hashing represents a *one-way function* that irreversibly transforms the source *data* used as input into a plain text output (i.e., the *hash* of *data*).

**Data Consistency**: A problem that could emerge adopting the proposed paradigm is certainly related to the consistency of the data stored on the blockchain [16,63,64]. In this sense, excessive network latency or the presence of malicious nodes could compromise the *chronological order* of *entities*’ registration. On the other hand, replay or tampering attacks [65] on transactions are natively prevented by the digital signature protocols of most existing blockchain systems [66].

However, regarding the chronological order, we remark that even a delayed (or missed) registration can generate, in some specific scenarios, significant limitations in the use of the *IoE* paradigm. Notably, several protocols [15,16,67,68] have been proposed in the literature to guarantee the consistency of transaction publication, mainly through incentive mechanisms parallel to those of the main consensus mechanism of the blockchain used. Although theoretically allowed, integrating such protocols with the *IoE* paradigm, as they are fundamentally domain-independent, represents a significant future development of this work to its more practical conceptualization.

### 6.2. IoE Technology Spread

As happened with other similar technologies, even in the proposed *IoE* one, the greatest obstacle to overcome is the spread across users of such a technology.

Although it is possible to create a new network of devices that operate according to the proposed *IoE* paradigm, we can substantially reduce this problem by integrating the *IoE* network into the existing *wireless-based* ones (e.g., *IoT* and *mobile*). This process, which allows us to maximize the *IoE* potential, can be facilitated by adopting several strategies, such as the following ones:

(i) Designing transparent and straightforward procedures of integration of the needed *IoE* functionalities in the existing e-health devices. This can be achieved, for instance, by integrating these as a *service* in the new devices, by recurring to a well-documented firmware/software upgrade process, or by making available an application (in those cases where the *trackers* or the *entities* are implemented in devices that allow us this solution, e.g., *smartphones*, *tablets*, etc.);
(ii) Making effective campaigns of information aimed to underline the advantages for each user that joins the *IoE* network, empathizing the gained opportunity to exchange data between a large community of users, a massive amount of valuable data that they can exploit in many contexts, such as that of *localization* taken into account in this paper;
(iii) Offering benefits to the users that join their devices to the *IoE* network as *trackers*, allowing the system to perform the *entity detection* and the *distributed-ledger registration* tasks. Such benefits could include the free use of some services related to the *IoE* network, such as, for instance, the services used for remote data storage.

As previously underlined, the exploitation of the mobile network contributes to impress a substantial acceleration to the spread of the *IoE* network, since such a network already involves an enormous number of potentially configurable devices, by recurring to simple applications, to operate according to the *IoE* paradigm. In this case, the information related to the geographic *location* of the *trackers* can be obtained by a local service (i.e., *GPS*) or by querying the mobile *cell* to which the e-health is connected. The sensors data related to the e-health side will be those available for that device; otherwise, this data will be absent.

The use case taken into account in this paper relies on the interaction between *entities* and *trackers*, implemented by using custom (e.g., wearable solutions) or standard (*IoT, smartphone*, and *tablet*) devices. However, the *IoE* potentiality could be improved by adding to the *IoE* network other classes of devices such as, for instance, *routers*, *access-points*, *hot-spots*, and many others. Although this type of expansion is potentially practicable, it requires an implementation effort more significant than needed by using the devices we considered in this paper.

**Business Models**: Some conclusive general observations are about exploiting the proposed *IoE* paradigm in the context of a hypothetical commercial scenario. From the point of view of a *Business-to-Business* (*B2B*) model, we can start by observing that many financial analysts underline that only the area related to the *IoT* has given rise to an interesting and profitable financial market, whose value in the next *5-10* years has been estimated around trillions of dollars [69].

Consequently, as a specialized sub-area of the *wireless-based* technologies market, the proposed *IoE* paradigm could offer new stimulating and profitable opportunities, considering that its applications involve a considerable number of customers, both private and commercial ones. To summarize, the activity core could be oriented towards developing *IoE* solutions for business customers, who in turn can offer this service to their customers, according to a *Business-to-Consumer* (*B2C*) model.

Such solutions involve both hardware and software aspects, from the hardware/software development of the *IoE* devices (e.g., *wearable devices*, *smartphone applications*, *vehicle equipment*, etc.) to the management of the needed services (e.g., *unique identifier distribution*, *remote storage*, etc). In some cases, these opportunities could be further expanded by defining and offering services in partnership with public or private investigative agencies (e.g., *security guards*, *local police*, etc.), giving rise to an attractive transversal market.

A *B2C* scenario could also include other services such as, for instance, the management of *entities* initially directly managed by customers or the development and commercialization of custom hardware and software solutions.

## 7. Conclusions

In this paper, we generalized and extended our novel paradigm, which we baptized *Internet of Entities* (*IoE*). First, we discussed the context in which we proposed our paradigm. Then, we formalized the notions of *entity* and *trackers*, providing also a timely definition of the interaction model and communication protocols between *entities*, *trackers*, and the blockchain. Subsequently, we performed a series of experiments aimed to verify and evaluate the practical feasibility of the proposed *IoE* paradigm in a real-world use case. We also identified specific heuristics and strategies for reconstructing the *entities* movements and, finally, we discussed the potential usage applications and future implementation of our paradigm.

The IoE paradigm joins the capabilities of the *wireless-based* environment with the certification capability provided by the blockchain-based distributed ledgers. It has two core components, *entities* and *trackers*, billions of new or already-existing devices that operate interchangeably across the its environment. Although such a paradigm exploits existing and widespread technologies, it offers a novel way to reliably trace the activity of people and objects in a certified and privacy-friendly way, producing valuable, exploitable, and investigative-valid data. In this sense, the concept of *robust network in its unstructured simplicity*, expressed by *Satoshi Nakamoto* during their *Bitcoin* formulation [2], well describes also the *Internet of Entities* network, whose capabilities are destined to grow, day after day, thanks to the continuous introduction of new wireless devices, which provide an ever-expanding coverage area.

Concluding, if, on the one hand, the proposed IoE paradigm can be easily implemented by exploiting existing technologies and infrastructures, on the other hand, it produces a series of advantages for the community, revealing the potential for growth in many real-world scenarios, such as that of the localization taken into consideration in this paper. 

## Figures and Tables

**Table 1:** Wireless technologies.

Wireless	Frequency	Data	Operative	Power	Security	Literature
Technology	Range	Rate	Range	Consumption	Protocols	Reference
**BLE**	2.4 GHz	1 MBps	15 ÷ 30 m	low	E0, Stream, AES-128	[49]
**6LoWPAN**	868 MHz ÷ 2.4 GHz	250 KBps	10 ÷ 100 m	low	AES	[50]
**Z-Wave**	868 MHz ÷ 908 MHz	40 KBps	30 ÷ 100 m	low	AES-128	[51]
**ZigBee**	2.4 GHz	250 KBps	10 ÷ 100 m	low	AES	[52]
**NFC**	868 MHz ÷ 902 MHz	106 ÷ 424 KBps	0 ÷ 1 m	Ultra-low	RC4	[53]
**RFID**	125 KHz ÷ 928 MHz	4 MBps	0 ÷ 200 m	Ultra-low	RSA, AES	[54]
**SigFox**	125 KHz ÷ 860 MHz	100 ÷ 600 Bps	10 ÷ 50 Km	low	no-specific	[55]
**2G/3G**	380 MHz ÷ 1.9 GHz	10 MBps	Several Kms	High	RC4	[56]

**Table 2:** Experimental results using BLE as wireless technology.

Experiment	Number	Number	Number	Average	Ethereum	Litecoin	Binance
	of	of	of	Communication Time	Average Cost per	Average Cost per	Average Cost per
Identifier	Entities	Trackers	Detections	per Tracker (s)	Tracker (USD)	Tracker (USD)	Tracker (USD)
1	6	2	3	7.88	11.10	0.02	0.41
2	6	4	7	9.19	12.95	0.03	0.47
3	6	6	10	8.75	12.33	0.03	0.45
4	8	2	5	13.13	18.50	0.04	0.68
5	8	4	10	13.13	18.50	0.04	0.68
6	8	6	14	12.25	17.27	0.04	0.63
7	10	2	6	15.75	22.20	0.05	0.81
8	10	4	14	18.38	25.90	0.06	0.95
9	10	6	19	16.63	23.43	0.05	0.86

**Table 3:** Experimental results using RFID as wireless technology.

Experiment	Number	Number	Number	Average	Ethereum	Litecoin	Binance
	of	of	of	Communication Time	Average Cost per	Average Cost per	Average Cost per
Identifier	Entities	Trackers	Detections	per Tracker (s)	Tracker (USD)	Tracker (USD)	Tracker (USD)
10	6	2	6	15.75	22.20	0.05	0.81
11	6	4	13	17.06	24.05	0.05	0.88
12	6	6	19	16.63	23.43	0.05	0.86
13	8	2	9	23.63	33.30	0.07	1.22
14	8	4	18	23.63	33.30	0.07	1.22
15	8	6	25	21.88	30.83	0.07	1.13
16	10	2	12	31.50	44.40	0.10	1.62
17	10	4	25	32.81	46.25	0.10	1.69
18	10	6	35	30.63	43.17	0.09	1.58

## Data Availability

Not applicable.
